# Responders to first-line osteoarthritis treatment had reduced frequency of hip and knee joint replacements within 5 years: an observational register-based study of 44,311 patients

**DOI:** 10.2340/17453674.2024.41011

**Published:** 2024-07-15

**Authors:** Kristin GUSTAFSSON, Anna CRONSTRÖM, Ola ROLFSON, Eva AGEBERG, Therese JÖNSSON

**Affiliations:** 1 Unit of Physiotherapy, Department of Health, Medicine and Caring Sciences, Linköping University, Linköping; 2 Department of Physiotherapy, Rehabilitation Centre, Ryhov County Hospital Jönköping, Jönköping; 3 Department of Health Sciences, Faculty of Medicine, Lund University, Lund; 4 Department of Community Medicine and Rehabilitation, Umeå University, Umeå; 5 Department of Orthopedics, Institute of Clinical Sciences, Sahlgrenska Academy, University of Gothenburg, Gothenburg; 6 Skane University Hospital, Orthopedics, Lund, Sweden

## Abstract

**Background and purpose:**

First-line treatment (education, exercise) for patients with hip and knee osteoarthritis (OA) aims to reduce pain and improve function. We aimed to compare progression to joint replacement within 5 years between responders and non-responders to first-line treatment for hip and knee OA, respectively.

**Methods:**

This observational study included data for 30,524 knee OA and 13,787 hip OA patients from the Swedish Osteoarthritis Register, linked with the Swedish Arthroplasty Register, Statistics Sweden, and the Swedish Prescribed Drug Register. The primary prognostic factor was change in pain between baseline and 3-month follow-up, measured on a numeric rating scale (0–10, best to worst) where an improvement of ≥ 2 was classified as responder and ≤ 1 as non-responder. The main outcome was progression to joint replacement surgery within 5 years, assessed using baseline adjusted multivariable Cox regression analyses.

**Results:**

At 5 years, in hip OA, 35% (95% confidence interval [CI] 32.2–37.2) of the responders and 48% (CI 45.9–49.5) of the non-responders and in knee OA 14% (CI 13.0–15.3) of the responders and 20% (CI 18.8–20.8) of the non-responders had progressed to joint replacement. Being a responder to the treatment was associated with having a lower probability of progression to surgery for both hip OA (hazard ratio [HR] 0.4, CI 0.4–0.5) and knee OA (HR 0.6, CI 0.5–0.6).

**Conclusion:**

Patients with hip or knee OA who experienced pain relief after a first-line OA treatment program were less likely to progress to joint replacement surgery.

Hip and knee osteoarthritis (OA) are among the leading causes of disability worldwide, affecting approximately 400 million people [1], and can lead to long-lasting pain, poor quality of life, and sick leave [2]. International guidelines recommend education, exercise, and weight control as first-line OA treatment [3,4]. If first-line OA treatment is not sufficiently effective, additional treatments like aids, passive treatments, pharmacological pain relief, and joint replacement surgery may be considered [3,4]. Several studies report that first-line treatment for OA improves pain, physical function, and quality of life, delays joint replacement, and changes patients’ willingness for surgery [5-8]. Despite these positive effects, only low proportions of patients are treated according to the guidelines before being referred for joint replacement [9,10].

Recently, Gustafsson et al. reported that 46% of those with hip OA and 20% of those with knee OA progress to joint replacement within 5 years after referral to a first-line treatment program for OA and that willingness for surgery was the strongest factor for progression to surgery [11]. First-line OA treatment aims to reduce symptoms, such as pain, and to educate and provide patients with tools that support them in self-management of OA. The goal is to achieve long-term lifestyle changes, particularly regarding physical activity and weight control, enabling the person to live a good and active life with OA [12]. However, it is still unclear if the change in symptoms (e.g., pain) after participating in first-line treatment for OA is associated with future progression to hip or knee replacement.

This register-based cohort study aimed to compare progression to joint replacement within 5 years between responders and non-responders to a first-line treatment program in patients with hip and knee OA, respectively.

## Methods

### Study design

We performed a longitudinal observational register-based study with prospectively collected data reported according to the Strengthening the Reporting of Observational studies in Epidemiology (STROBE) guidelines [13].

### Data sources and participants

The Swedish Osteoarthritis Register (SOAR) is a national quality register that follows and evaluates patients with OA who participate in a standardized 3-month first-line OA treatment program. Patients referred to the program are identified in primary health care and are assessed as not eligible for surgery. The program includes patient education and exercise and is delivered following national and international OA treatment guidelines. Today, SOAR comprises data from more than 800 different physiotherapy units in primary health care in Sweden [12,14], with 86% coverage (the number of rehabilitation units that offer the standardized first-line OA treatment program and report to SOAR) and 72% completeness (the number of patients who participate in the standardized first-line OA treatment program and report to the SOAR) [15]. Patient-reported questionnaires including sex, age, BMI, Charnley score, willingness for surgery, pain intensity, pain frequency, and health-related quality of life were registered at baseline, and at follow-up at 3 and 12 months, and physiotherapist-reported questionnaires including diagnoses were registered at baseline and compliance with the treatment at 3 months [12].

Patients are eligible for the first-line OA treatment program if they have a clinical and/or radiographically diagnosed OA [12], in accordance with the Swedish National Board of Health and Welfare [4]. Their recommendation is that OA at this stage of the disease should be clinically diagnosed, based on a clinical assessment through an overall judgment of patient history, symptoms, and clinical findings [4]. This is also based on the established discrepancy between symptoms and structural changes identified by radiography [16,17] and the fact that awaiting radiographically detectable changes could delay initiating first-line treatment [2,4]. Patients are not eligible for the program if they have: joint problems for any other reason (e.g., sequel hip fractures, chronic widespread pain, inflammatory joint diseases, or cancer); received a joint replacement within the past 12 months; undergone other surgeries of the hip or knee joint within the past 3 months; and/or were not able to read or understand Swedish [12].

Data on all patients in the SOAR between 2008 and 2016 (n = 72,069) were merged with the Swedish Arthroplasty Register, to identify joint replacements due to OA (replacement surgery due to other diagnoses were excluded) performed after date of baseline during the study period. Individual-level data on socioeconomic factors was then added from the Longitudinal Integration Database for Health Insurance and Labour Market Studies (LISA) at Statistics Sweden and on comorbidities from the Swedish Prescribed Drug Register governed by the National Board of Health and Welfare, Sweden [18]. Patients who were registered in the SOAR with data from both patient and physiotherapist questionnaires at baseline and at 3-month follow-up (±2 months), including complete reporting of pain on both occasions, were included in the study. The linkage between registers was performed using the unique 10-digit personal identity numbers assigned to all Swedish residents at birth or immigration. The Swedish Arthroplasty Register is a national quality register that records 97–98% of all hip and knee replacements in Sweden [19], while Statistics Sweden and the National Board of Health and Welfare are nationwide, mandatory, and government-maintained registers. Detailed information regarding the data sources and the merging of the different registers has been described in a study protocol [18].

### Prognostic factor

The primary prognostic factor was change in pain between baseline and the 3-month follow-up, measured with a numeric rating scale (NRS). The NRS comprises an 11-point scale where 0 indicates no pain and 10 indicates the worst possible pain during the last week [20]. Patients with ≥ 2 step improvement in pain on NRS were classified as responders, while those with ≤ 1 step improvement (or no change/deterioration in pain) were classified as non-responders [21].

### Outcomes

The main outcome was progression to hip or knee joint replacement within 5 years after participation in the first-line OA treatment program (dichotomous [yes/no]). For patients with hip OA, the outcome was total hip replacement due to OA. For patients with knee OA, the outcome was total or partial knee replacement surgery due to OA.

### Covariates

Covariates were baseline age, sex, body mass index (BMI), pain frequency, pain intensity, willingness for surgery, Charnley score, health-related quality of life, participation in supervised exercise, comorbidities, and disposable income, all previously reported as covariates for surgery in this population [11,22]. BMI was calculated from self-reported weight and height. Pain frequency was assessed by the question: “How often do you have pain in your knee/hip,” with 5 possible answers: never, every month, every week, every day, or all the time. Pain intensity was assessed with the NRS (0–10) [20]. Willingness for surgery was assessed by the question: “Are your knee/hip symptoms so severe that you wish to undergo surgery?” (yes/no). Musculoskeletal comorbidities were assessed with Charnley score, which is a patient-reported classification of musculoskeletal impairment, divided into (A) unilateral hip or knee OA, (B) bilateral hip or knee OA, or (C) multiple joint OA or presence of another condition that affects the ability to walk [23]. Health-related quality of life was assessed using the EQ-5D-3L index, developed by the EuroQoL group [24]. The United Kingdom value set for weighting was used, which ranges from a minimum level of –0.59 to a maximum of +1.00, with a higher value indicating better health [25]. Participation in supervised exercise was reported by the physiotherapist responsible for the treatment. Comorbidities were summarized with the RxRisk Index, which identifies 43 different conditions [26], through data on the Swedish Prescribed Drug Register of the National Board of Health and Welfare. In the present study, the 2 conditions “inflammation/pain” (treated with anti-inflammatory medication) and “pain” (treated with narcotics) were considered as index conditions and thereby excluded from the calculations. Disposable income was extracted from Statistics Sweden.

### Statistics

All analyses were stratified based on the most affected joint (hip and knee). To estimate the cumulative rate of hip or knee replacement based on categorizing change in pain on NRS into responders or non-responders, Kaplan–Meier survival analyses with 95% confidence intervals (CI) were calculated as years from baseline to the time of event. Study participants who did not undergo a hip or knee replacement surgery were censored 5 years after baseline, at time of death, or at the end of 2016, whichever occurred first. The estimated rates of replacements for patients with hip or knee OA were reported at 1 and 5 years, respectively. Comparison between responders and non-responders of progression to surgery within 5 years was also presented as risk ratio (RR) with 95% CI. Multivariable Cox proportional hazards regression analyses were used to investigate the effect of change in pain on progression to hip and knee replacement. Hazard ratios (HR) with 95% CI were reported, where the category “non-responders” was used as reference. The models were reported with both crude and adjusted data (baseline age, sex, BMI, pain frequency, pain intensity, Charnley score, comorbidities, willingness for surgery, health-related quality of life, participation in supervised exercise, and income). The proportional hazard assumption was checked graphically. All statistical analyses were performed with IBM SPSS Statistics for Windows, v27.0 (IBM Corp, Armonk, NY, USA).

### Ethics, data sharing, funding, and disclosures

This study was approved by the Regional Ethical Review Board in Gothenburg, Sweden (16-03-2017, entry number 1059–16) and registered at clinicaltrials.gov (NCT03438630). Data used in the study is governed by Västra Götalandsregionen and the authors are not permitted to share the data. Data can be made available from Registercentrum Västra Götalandsregionen for researchers who meet the criteria for access to confidential data according to Swedish law (contact artrosregistret@registercentrum.se). This study was supported by funding from Johan and Greta Kock’s foundation, PI (TJ), AFA Insurance Sweden (160176, PI (OR)), Futurum – the Academy for Health and Care, Region Jönköping County Sweden (936222, PI (KG)), the Medical Research Council of Southeast Sweden (744201, PI (KG)), Governmental Funding of Clinical Research within the National Health Service (2018-Projekt0080 and 2022-Projekt0050, PI (EA), and the ALF-agreement project ALFGBG-965217 (OR). The authors declare no conflict of interest for this work. Complete disclosure of interest forms according to ICMJE are available on the article page, doi: 10.2340/17453674.2024.41011

## Results

### Study participants

**Figure 1:**
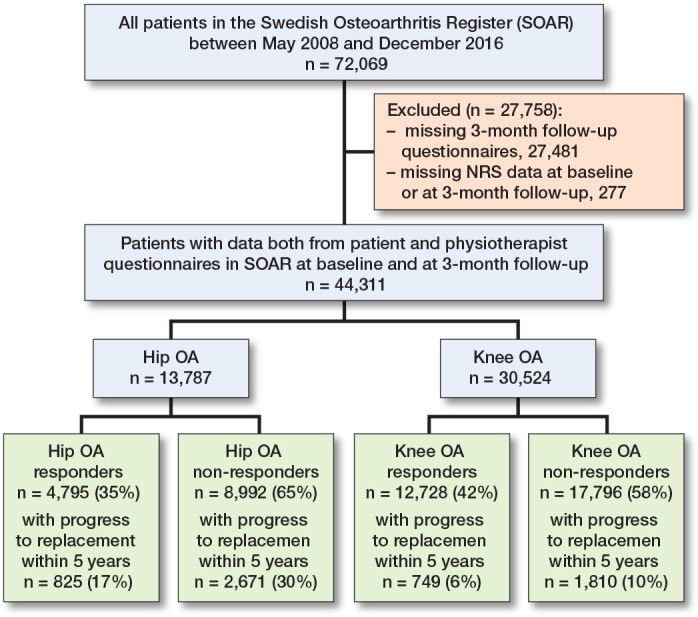
Flowchart of the study population.

44,311 patients with hip (n = 13,787) or knee (n = 30,524) OA were included in the study (Figure 1). In patients with both hip and knee OA, patients who were categorized as responders were, on average, 1 year younger. There were slightly more women among the responders, and they reported, on average, higher pain intensity and more frequent pain at baseline compared with the non-responders. Among patients with knee OA, the responders had less musculoskeletal comorbidity (assessed with Charnley Class), while the differences were less pronounced among those with hip OA (Table). Among those with hip OA, 3,496 patients (25%) underwent a hip replacement, while among patients with knee OA, 2,559 patients (8%) underwent a knee replacement, during the study period. Median time to surgery was 379 days (interquartile range [IQR] 233–643 days) for those with hip OA, and 615 days (IQR 372–958 days) for those with knee OA.

**Table:** Baseline characteristics of patients with hip and knee OA, reported for all and according to categories for change in pain

	Total	Missing	Responders^a^	Non-responders^b^	Difference between responders and non-responders (CI)	Excluded
**Hip OA**						
N (%)	13,787		4,795 (35)	8,992 (65)		8,916
Age at baseline, mean (SD)	67 (9)	0	67 (9)	68 (9)	–0.7 (–1.0 to –0.4)	67 (10)
Women, %	68	0	70	68	2 (1 to 4)	67
Disposable income (US$ x 10^3^), median (IQR)	20.5 (15.6–27.9)	2	20.6 (15.6–27.9)	20.4 (15.6–27.8)		20.2 (15.2–27.8)
Body mass index, mean (SD)	27.0 (4.4)	226	27.0 (4.3)	27.0 (4.5)	–0.01 (–0.17 to 0.15)	27.3 (4.9)
Comorbidities^c^, median (IQR)	3 (1–5)	0	3 (1–5)	3 (1–5)		3 (1–5)
Charnley classification^d^, %		0				
A	38		37	39	–1 (–3 to 0)	37
B	10		11	10	0 (–1 to 1)	10
C	52		52	51	1 (–1 to 3)	53
Willingness for surgery (yes), %	28	124	25	30	–5 (–7 to –4)	35
Pain intensity (NRS, 0–10), mean (SD)	5.4 (1.9)	0	6.2 (1.6)	5.0 (2.0)	1.2 (1.1 to 1.2)	5.7 (2.0)
Pain frequency (every day/all the time), %	84	39	86	83	3 (1 to 4)	86
EQ-5D index, mean (SD)	0.61 (0.23)	255	0.60 (0.24)	0.62 (0.23)	–0.02 (–0.03 to –0.01)	0.57 (0.26)
Participation in supervised exercise, %		498				
≥ 10 sessions	31		32	30	2 (–0 to 3)	
7–9 sessions	11		11	11	0 (–1 to 1)	
1–6 sessions	17		16	17	–1 (–2 to 1)	
0 sessions	41		41	42	–1 (–3 to 1)	
Hip replacement within 5 years^e^, n (%)	3,496 (25)	0	825 (17)	2,671 (30)	–13 (–14 to –11)	2,658 (30)
**Knee OA**						
N (%)	30,524		12,728 (42)	17,796 (58)		18,842
Age at baseline in years, mean (SD)	66 (9)	0	66 (9)	67 (9)	–0.7 (–0.9 to –0.4)	66 (10)
Women, %	70	0	71	69	2 (1 to 3)	68
Disposable income (US$ x 10^3^), median (IQR)	21.1 (15.9–28.2)	0	21.2 (16.1–28.2)	20.9 (15.7–28.2)		20.6 (15.3–30.0)
Body mass index, mean (SD)	28.4 (5.0)	560	28.5 (5.1)	28.3 (4.8)	0.13 (0.18 to 0.25)	28.7 (5.2)
Comorbidities c, median (IQR)	3 (1–5)	0	3 (1–5)	3 (1–5)		3 (1–5)
Charnley classification d, %		0				
A	38		41	36	5 (4 to 6)	38
B	23		23	24	–1 (–2 to –0)	22
C	39		36	40	–4 (–5 to –3)	40
Willingness for surgery (yes), %	23	300	23	23	0 (–1 to 1)	29
Pain intensity (NRS), mean (SD)	5.2 (2.0)	0	6.0 (1.6)	4.7 (2.0)	1.3 (1.3 to 1.4)	5.5 (2.0)
Pain frequency (every day/all the time), %	82	86	85	79	6 (5 to 7)	83
EQ-5D index, mean (SD)	0.64 (0.22)	532	0.63 (0.23)	0.65 (0.22)	–0.03 (–0.03 to –0.02)	0.61 (0.25)
Participation in supervised exercise, %		1,150				
≥ 10 sessions	30		32	29	3 (2 to 4)	
7–9 sessions	12		12	12	0 (–1 to 1)	
1–6 sessions	17		16	17	–1 (–2 to -0)	
0 sessions	41		40	42	–2 (–3 to –1)	
Knee replacement within 5 years^e^, n (%)	2,559 (8.4)	0	749 (5.9)	1,810 (10)	–4 (–5 to –4)	1.741 (9.2)

a Responders are defined as ≥ 2-step improvement in pain on the NRS.

b Non-responders are defined as ≤ 1-step improvement in pain (including no change/deterioration) on the NRS.

c Summarized with the RxRisk Index.

d Charnley classification: A (unilateral hip or knee OA), B (bilateral hip or knee OA), and C (multiple joint OA or presence of another condition that affects the ability to walk).

e Rates and proportion of each group that progressed to hip respective knee replacement within 5 years.

OA = osteoarthritis; SD = standard deviation; CI = confidence interval; IQR = interquartile range; NRS = numeric rating scale.

The patients who were excluded from the analyses due to missing 3-month data had similar baseline characteristics to the included patients, except for the proportion that reported willingness for surgery at baseline and progression to joint replacement (Table).

### Progression to joint replacement

**Figure 2:**
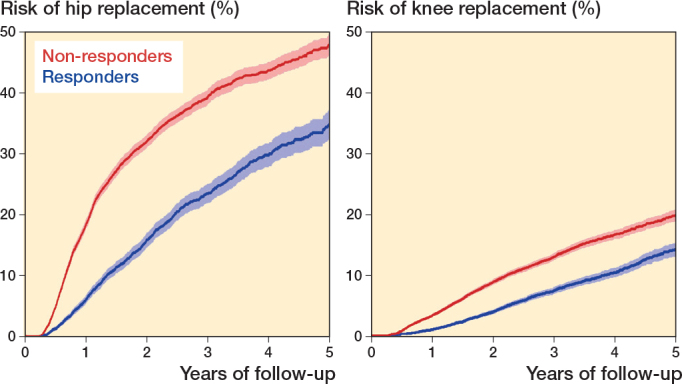
A Kaplan–Meier graph showing the estimated proportion of patients with hip OA (left panel) that did proceed to hip replacement and knee OA (right panel) that did proceed to knee replacement, after participation in a first-line OA treatment program, with 95% confidence intervals and stratified by change (non-responders ≤ 1 step / responders ≥ 2 steps) in pain on a numeric rating scale (NRS).

For hip OA, the Kaplan–Meier estimates showed that 6% (CI 5.4–6.9) of the responders and 19% (CI 17.7–19.5) of the non-responders progressed to a joint replacement within 1 year after participating in a first-line OA treatment program. The corresponding rates at 5 years were 35% (CI 32.2–37.2) and 48% (CI 45.9–49.5) (Figure 2). For knee OA, 1% (CI 0.9–1.2) of the responders and 3% (CI 3.0–3.6) of the non-responders progressed to joint replacement at 1 year. The corresponding rates at 5 years were 14% (CI 13.0–15.3) and 20% (CI 18.8–20.8) (Figure 2). The RR for progression to replacement surgery within 5 years was 0.6 (CI 0.6–0.7) for hip OA, and 0.7 (CI 0.6–0.7) for knee OA.

The Cox regression analyses showed that being a responder was associated with a decreased probability of progression to surgery compared with those who did not respond to the first-line OA treatment program. This was true for hip OA (crude HR 0.5, CI 0.5–0.5, adjusted HR 0.4, CI 0.4–0.4), and knee OA (crude HR 0.6, CI 0.5–0.6, adjusted HR 0.4, CI 0.4–0.5).

## Discussion

This is the first large-scale register-based study including more than 44,000 patients with hip and knee OA exploring this association between change in pain after a first-line OA treatment program and progression to joint replacement up to 5 years post program participation. We aimed to compare progression to joint replacement between responders and non-responders to first-line treatment for hip and knee OA, respectively. We showed that being a responder to the first-line OA treatment program was associated with less probability of progression to joint replacement within 5 years compared with being a non-responder. This association was true for both hip OA and knee OA.

The findings of our study are aligned with a recently published registry-based study conducted by Ackermann et al. that analyzed data from 9,000 patients diagnosed with hip or knee OA in Denmark [27]. Similar to our results, they demonstrated that patients who responded to treatment had a significantly lower probability of progressing to surgery compared with non-responders, and they found that patients with hip OA tended to undergo surgery more frequently than patients with knee OA. While we found a difference in time to surgery between patients with hip OA and knee OA (379 days vs 615 days), the Danish study did not (356 days vs 357 days) [27]. However, it is important to compare these results with caution as the studies calculate time to surgery from different starting points. Additionally, they revealed that improvements in hip- or knee-related quality of life and arthritis self-efficacy (pain) following a first-line treatment program were significantly associated with lower likelihood of progression to joint replacement within 2 years [27]. The results from our study demonstrate similar findings to those reported by Ackermann et al. in a smaller population. Furthermore, we indicate that these results appear to persist for up to 5 years after first-line treatment.

The effect of first-line treatment programs on progression to replacement in hip and knee OA has also been evaluated in randomized trials. Long-term follow-up from Norway indicates that the need for total hip replacement might be reduced by 44% following a first-line treatment program in patients with hip OA without simultaneously reporting any pain reduction [6]. A secondary analysis of 2 randomized trials in Denmark showed that 2 out of 3 patients with knee OA, who were already eligible for total knee replacement, could delay surgery for at least 2 years when following a first-line OA treatment program [5]. The results from the present study and previous studies [5,6,27] support the importance of patients participating in a first-line OA treatment program before being referred for consideration of joint replacement. Healthcare providers stand to gain valuable insights from both our current study and prior research on how the effectiveness of initial treatments influences the likelihood of surgery. These findings can inform discussions with patients regarding the continuation of first-line treatment or referral to an orthopedic clinic for consideration of surgery. Furthermore, our findings may help improve the selection of suitable candidates for surgery and identify optimal timing for referral, thereby alleviating the burden on orthopedic clinics.

### Limitations and strengths

***Limitations.*** First, the observational nature of this study, i.e., without a control group, does not allow us to establish causality on the association between the results of a first-line OA treatment program and progression to joint replacements. Second, the study is conducted in a real-world setting, so we cannot be entirely sure of the classification used by the healthcare professionals in primary care when diagnosing OA. Furthermore, the dropout rate (patients who were excluded due to missing follow-ups) was high. Nevertheless, the baseline characteristics of the excluded were similar to those of the patients included in the study. The exception was that a higher proportion of those excluded expressed a willingness for surgery at baseline and progressed to joint replacement within 5 years, particularly those with hip OA. The reasons for dropout are not known, but a contributing factor could be progression to joint replacement before the 3-month follow-up, as previous research has shown that a decision to have joint replacement appears to be highly affected by the patient’s willingness for surgery [11,28]. Third, we observed a difference in pain intensity at baseline, with responders having, on average, higher baseline pain than non-responders to the first-line OA treatment program. Therefore, we adjusted all analyses for baseline pain to minimize the regression-to-the-mean effect on the results. Fourth, we do not have information regarding when patients were referred to this first-line treatment program during the disease course, but they have most likely sought primary care at an earlier stage of the disease. Furthermore, we do not have information on how well the patients in the study adhered to the exercises after finishing the program. Finally, it is important to recognize that healthcare systems may vary between countries, which could potentially limit the generalizability of our findings beyond Sweden.

***Strength.*** The present study included more than 44,000 patients cross-linked from well-validated registers.

### Conclusion

Patients with hip or knee OA who experience pain relief after a first-line OA treatment program were less likely to progress to joint replacement surgery.

***Perspective.*** Patients should be offered support with their OA management and participation in structured first-line treatment during the course of the disease. However, further research is needed to investigate whether initiating the program at different stages of OA, as well as exercise adherence over time, has any impact on the likelihood of future joint replacement surgery.

## Notes

KG had full access to all of the data in the study and takes responsibility for the integrity of the data and the accuracy of the data analysis including statistical analysis. Drafting of the manuscript: KG, AC, and TJ. Concept and design, acquisition, analysis, or interpretation of data, and critical revision of the manuscript for important intellectual content: all authors.

The authors would like to acknowledge all participating patients, healthcare staff reporting data to the Swedish Osteoarthritis Register, and the Swedish Arthroplasty Register.

Handling co-editors: Keijo Mäkelä and Robin Christensen

*Acta* thanks Jeppe Lange and Einar Andreas Sivertsen for help with peer review of this manuscript.
